# Novel Strategies to Address Critical Challenges in Pancreatic Cancer

**DOI:** 10.3390/cancers14174115

**Published:** 2022-08-25

**Authors:** Jisce R. Puik, Rutger-Jan Swijnenburg, Geert Kazemier, Elisa Giovannetti

**Affiliations:** 1 Department of Surgery, Amsterdam UMC Location Vrije Universiteit Amsterdam, De Boelelaan 1117, 1081 HV Amsterdam, The Netherlands; 2 Cancer Center Amsterdam, Imaging and Biomarkers, 1081 HV Amsterdam, The Netherlands; 3 Department of Surgery, Amsterdam UMC Location University of Amsterdam, 1105 AZ Amsterdam, The Netherlands; 4 Cancer Pharmacology Lab, Associazione Italiana per la Ricerca sul Cancro (AIRC) Start-Up Unit, Fondazione Pisana per la Scienza, University of Pisa, 56126 Pisa, Italy

Whereas mortality rates improved for breast and prostate cancer as a result of successful tumour biology-based therapies and biomarkers, mortality rates for pancreatic cancer patients remained stable [1,2]. Incidence is growing, and 5-year survival rates are approximately 10% [1,3]. As such, pancreatic cancer is set to surpass breast and colorectal cancer to become the second leading cause of cancer-related death by 2030 [1,2]. Its aggressive nature and highly complex tumour microenvironment (TME) form major obstacles in current diagnostic and therapeutic strategies. This Special Issue of *Cancers* discusses novel, promising approaches in the fields of diagnosis, overcoming resistance to chemotherapy and new therapeutic targets.

One of the major issues for patients with pancreatic ductal adenocarcinoma (PDAC) is the lack of early and accurate diagnosis. Patients with PDAC in the pancreatic head may only present with complaints of jaundice at advanced tumour stages. Furthermore, as described by Boyd et al., current diagnostic strategies seem to be unable to accurately differentiate PDAC from distal cholangiocarcinoma and malignant from benign disease in the pancreatic head [4]. The only validated biomarker that is currently used in the clinic is carbohydrate antigen 19-9 (CA19-9). CA19-9 levels are typically elevated under circumstances of hyperbilirubinemia, which can also develop due to benign biliary tree obstructions. Boyd et al. assessed and optimized the clinical potential of CA19-9 by studying CA19-9, bilirubin and their ratio CA19-9/(bilirubin^−1^) in 232 patients with hepatobiliary disease [4]. Interestingly, their ‘Model Ratio’ showed superior accuracy when detecting PDAC. Similarly, Mantini et al. stress that for a specific diagnosis of PDAC, especially when using machine learning, it is of great importance to use an integrative approach [5]. By using gene-ontology mining of -omics data, they demonstrated that biological mechanisms in circulating platelets, such as active RNA processing and differential regulation of SPARC, are essential for biomarker discovery.

Another emerging strategy is diagnosis through tumour-specific radiolabelled tracers. ^18^F-Fluorodeoxyglucose Positron Emission Tomography (^18^F-FDG PET) is an imaging modality that relies on the metabolism of radiolabelled glucose by cells. Cancer cells often metabolize more glucose and, hence, show higher tracer uptake. The deployment of ^18^F-FDG PET, however, is limited for patients with PDAC because typically 90% of PDAC tumour volume consists of dense stromal reaction and only a small percentage of tumour cells [6]. Moreover, higher glucose uptake can also be present in inflammatory diseases, including pancreatitis. In their review, Poels et al. suggest that Fibroblast Activation Protein Inhibitor (FAPI) and Prostate-Specific Membrane Antigen (PSMA) are highly promising novel tracer targets [7]. PSMA is a transmembrane protein that not only seems to be present in prostate cancer cells but also in tumour-associated neovasculature [7]. When comparing PDAC with pancreatitis or healthy tissue using immunohistochemistry, the expression of PSMA was high and low (H-score 0), respectively [8]. Hopefully, PSMA expression can distinguish PDAC from pancreatitis when using the PSMA PET/CT. The latter is currently used globally for staging prostate cancer and the detection of recurrence [9]. Therefore, when proven effective for PDAC, this tracer can be readily available for clinical implementation.

Of note, not only are these tumour-targeted imaging tracers important for diagnostic purposes, they can also prove pivotal as radiopharmaceutical carriers for targeted therapy [7,10]. In a phase III trial, ^177^Lu-PSMA-617 treatment was compared to standard care in 831 patients with highly lethal castration-resistant prostate cancer [11]. Compared to standard care, it showed better progression free (8.3 versus 3.4 months, respectively; *p* < 0.001) and overall survival (15.3 versus 11.3 months, respectively; *p* < 0.001), as well as favourable secondary outcomes [11]. In March 2022, ^177^Lu-PSMA-617 treatments were approved by the FDA for this indication [12]. Radiolabelled tracers have already been shown to be effective and safe in patients with prostate cancer. Even though research studies on its applications in pancreatic cancer are still in their infancy, radiolabelled tracers could prove useful in diagnosing PDAC and carrying medicine or sensitizers to the tumour.

Finding strategies to predict treatment responses and improve therapeutic effects is essential for PDAC. Tumour heterogeneity is very high among patients [6]. Therefore, personalised treatment based on tumour genotype and phenotype could revolutionize clinical care. As described by Robatel et al. in their review, stratifying patients based on RNA transcriptional analyses has led to two suggested subtypes: basal-like and classical PDAC. Patients with the classical subtype showed improved responses to FOLFIRINOX [6,13]. Moreover, patient-derived organoid cultures could form another strategy for patient stratification. They reflect histopathologic tumour features and contain patient-specific protein markers. Response to FOLFIRINOX or gemcitabine can be predicted using in vitro organoids in xenografts [6]. Robatel et al. also discusses that a single therapy, such as FOLFIRINOX, only shows limited effects due to tumour heterogeneity and resistance to chemotherapy [6]. A combination therapy would be more likely to elicit clinical benefits.

One of the major drivers in chemoresistance is Epithelial to Mesenchymal Transition (EMT) [14]. This is the process of transdifferentiation from the epithelial to a more mesenchymal-like types of cells. As Palamaris et al. describe, such mesenchymal features contribute to invasion, vascular extravasation, dissemination and drug resistance [14]. Since it is a multistep process that includes many signalling pathways, it offers numerous targets for therapy. These include TGF-β, IL-6, IL-1 and Hedgehog [14]. Combination therapies with chemotherapy and inhibitors of these targets are currently being studied in clinical trials for clinical applications [14]. To identify and quantify proteins associated with drug resistance, mass spectrometry-based proteomics has demonstrated to be a useful tool [15]. Using this tool for stable isotope labelling by amino acids in cell culture (SILAC)-based quantitative proteomics analysis, Kim et al. investigated which proteins are associated with oxaliplatin resistance in PANC-1 cells [15]. They identified 107 proteins (*p* ≤ 0.05), including myristoylated alanine-rich C-kinase substrates and WNTless homoloog proteins. The siRNA-mediated suppression of these two proteins also improved oxaliplatin sensitivity, underlining their involvement in chemotherapeutic responses.

Another method for improving chemosensitivity in PDAC, especially considering its challenging TME as a chemotherapeutic obstacle, is to gain control over drug-release sites and time [16]. Iacobazzi et al. focused on a double targeting strategy: pH and PDAC-specific receptor uPAR [16]. They developed pH-responsive polymeric micelles using a microfluidic-assisted preparation. To improve the selective targeting of PDAC, they functionalized micelles with a ligand for the uPAR receptor. Loaded with gemcitabine, this drug delivery system was tested in pancreatic cancer models that were co-cultured with cancer associated fibroblasts (CAFs). It showed increased apoptosis and arrest of the cell cycle, encouraging further in vivo studies [16].

Understanding the molecular mechanisms of oncogenic processes and interactions can lead to novel targets for targeted therapy. Targeted therapeutic approaches such as monoclonal antibodies to prevent HER2 interaction or tyrosine kinase inhibitors to block the phosphorylation of HER2 have not been successful and quickly result in resistance [17]. Stoup et al. studied the MUC4, which is already present in early stages of carcinogenesis, and MUC4/HER2 interaction for an alternative approach to target HER2 driven malignancy. The EGF domains of MUC4 showed an important role in HER2 binding affinity and tumour growth activity [17]. Therefore, MUC4_EGF_ domains could be of therapeutic value. Another promising novel therapeutic target that is proposed in the review of Wijnen et al. is cyclin-dependent kinase 1 (CDK1) [18]. CDK1 is involved in cell cycle progression by regulating the G2/M cell cycle checkpoint, and it promotes proteins that contribute to the formation of cancer stem cells (CSCs). Blockage of CDK1 resulted both in G2/M-mediated cell cycle arrest and apoptosis, as well as the inhibition of the clonogenic potential of CSCs by inducing cell differentiation [18]. Various preclinical studies have shown promising results. For example, treatments with Indox and 5MeOIndox, CDK-1 inhibitors significantly reduced tumour growth and weight in mice [18]. Moreover, the combination of CDK-1/2/4/5 inhibitors milciclib and gemcitabine in a phase I dose-escalation study in patients with refractory cancer suggested that the treatment was well tolerated, that there was minimal toxicity and that there seemed to be some clinical benefits [18]. These studies suggest that CDK1 could play a role in therapeutics when combined with ionising radiation therapy and DNA-damaging chemotherapy [18]. Evidently, clinical trials are warranted to evaluate and confirm the clinical use of CDK-1.

As a distinct alternative to directly targeting tumour cells, various studies are being conducted that evaluate the TME as a therapeutic target [19]. The TME of PDAC consists of dense, fibrous tissue with stromal cells, including both tumour-associated macrophages (TAMs) and CAFs. Malik et al. studied CXCL12, which is a chemokine that is secreted by these CAFs [19]. It promotes many carcinogenic signalling pathways, contributes to immune evasion, and promotes chemoresistance [19]. Thus, the inhibition of CXCL12 could possibly promote the effectiveness of immune therapy or chemotherapy. In *KPC* mouse models, targeting fibroblast activation protein-α (FAP^+^) CAFs that produced CXCL12 caused the suppression of antitumor immunity. When combined with previous targeted approaches, including CXCR4 inhibitor AMD3100 and anti PD-L1, it resulted in a synergistic response with tumour regression. This CXCL12/CXCR4 axis is also notorious for promoting gemcitabine resistance. Again, treatments with AMD3100 resulted in sensitized PDAC cells in vitro and in vivo [19]. Malik et al. suggest directly targeting CXCL12 through CXCL12 antagonists (NOX-A12) or oral farnesyl transferase inhibitor tipifarnib. However, further preclinical studies of these inhibitors are required.

Unlike previous mentioned strategies, it is hypothesized that it is also possible to target tissue injury repair mechanisms after another initial treatment [20]. In particular, Sugyo et al. hypothesized that refractory tumours that have received radiation therapy induce repair processes that could be targeted with additional therapy [20]. They selected an extracellular matrix glycoprotein that is upregulated specifically in tissue repair, tenascin-C (TNC), as repair-associated target. They developed three antibodies that recognized both human and murin TNCs and radiolabelled these with ^111^In. TNC expression was assessed using immunohistochemical staining and using single-photon emission computed tomography with computed tomography (SPECT/CT) studies. BxPC-3 pancreatic cancer xenografts were used as tumour model and initially treated with X-irridiaton or no X-irridation. Biodistribution studies showed higher tumour uptake of antibody 3–6. This was confirmed by SPECT/CT results. Therefore, antibody 3–6 could be a potential therapeutic strategy.

To conclude, this Special Issue of *Cancers* includes a collection of articles discussing some of the major challenges that we are facing in caring for pancreatic cancer patients as well as the latest strategies that have been deployed to overcome these. An important message emerging from all these studies is that more research studies and collaborations between different working fields are essential to change the prognosis of our PDAC patients.
